# A living cell quartz crystal microbalance biosensor for continuous monitoring of cytotoxic responses of macrophages to single-walled carbon nanotubes

**DOI:** 10.1186/1743-8977-8-4

**Published:** 2011-01-25

**Authors:** Gang Wang, Abiche H Dewilde, Jianping Zhang, Anoop Pal, Malavika Vashist, Dhimiter Bello, Kenneth A Marx, Susan J Braunhut, Joel M Therrien

**Affiliations:** 1 Department of Biological Sciences, University of Massachusetts Lowell, One University Ave., Lowell, MA, 01854, USA; 2 Department of Chemistry, University of Massachusetts Lowell, One University Ave., Lowell, MA, 01854, USA; 3 Department of Work Environment, University of Massachusetts Lowell, One University Ave., Lowell, MA, 01854, USA; 4 Department of Electrical and Computer Engineering, University of Massachusetts Lowell, One University Ave., Lowell, MA, 01854, USA

## Abstract

**Background:**

Numerous engineered nanomaterials (ENMs) exist and new ENMs are being developed. A challenge to nanotoxicology and environmental health and safety is evaluating toxicity of ENMs before they become widely utilized. Cellular assays remain the predominant test platform yet these methods are limited by using discrete time endpoints and reliance on organic dyes, vulnerable to interference from ENMs. Label-free, continuous, rapid response systems with biologically meaningful endpoints are needed. We have developed a device to detect and monitor in real time responses of living cells to ENMs. The device, a living cell quartz crystal microbalance biosensor (QCMB), uses macrophages adherent to a quartz crystal. The communal response of macrophages to treatments is monitored continuously as changes in crystal oscillation frequency (Δf). We report the ability of this QCMB to distinguish benign from toxic exposures and reveal unique kinetic information about cellular responses to varying doses of single-walled carbon nanotubes (SWCNTs).

**Results:**

We analyzed macrophage responses to additions of Zymosan A, polystyrene beads (PBs) (benign substances) or SWCNT (3-150 μg/ml) in the QCMB over 18 hrs. In parallel, toxicity was monitored over 24/48 hrs using conventional viability assays and histological stains to detect apoptosis. In the QCMB, a stable unchanging oscillation frequency occurred when cells alone, Zymosan A alone, PBs alone or SWCNTs without cells at the highest dose alone were used. With living cells in the QCMB, when Zymosan A, PBs or SWCNTs were added, a significant decrease in frequency occurred from 1-6 hrs. For SWCNTs, this Δf was dose-dependent. From 6-18 hrs, benign substances or low dose SWCNT (3-30 μg/ml) treatments showed a reversal of the decrease of oscillation frequency, returning to or exceeding pre-treatment levels. Cell recovery was confirmed in conventional assays. The lag time to see the Δf reversal in QCMB plots was linearly SWCNT-dose dependent. Lastly, the frequency never reversed at high dose SWCNT (100-150 μg/ml), and apoptosis/necrosis was documented in conventional 24 and 48 hr-assays.

**Conclusion:**

These data suggest that the new QCMB detects and provides unique information about peak, sub-lethal and toxic exposures of living cells to ENMs before they are detected using conventional cell assays.

## Background

### I. The challenge by nanotechnologies to nanotoxicology is combinatorial in nature: the expansion and variety of ENMs

Commercialization of ENMs is expanding quickly, with more than 2400 different nanoproducts already in commerce [1], outpacing our ability to conduct toxicological evaluations for human safety prior to large scale use of ENMs. Thus, there is a urgent need for the development of high-rate toxicity screening approaches [2,3]. At present, cellular assays remain the predominant toxicity testing platform for ENMs [4]. Common to cellular test systems is the use of various cell lines, single dosing at a range of concentrations and monitoring of a limited set of toxicological endpoints at discrete time intervals. Additionally, cellular testing relies heavily on organic dyes, labels and spectrophotometric techniques (ultraviolet or fluorescence measurements). Such protocols have several technical drawbacks, including: i) interference of the label by ENMs via absorption of detection dyes onto the large surface area of ENMs [5-8]; and ii) light scattering and/or quenching of fluorescence used in detection in the assay by the ENMs. In addition these types of protocols may be inadequate in detecting the mechanism of ENM toxicity because: i) discrete monitoring of toxic endpoints at particular time intervals may miss the recovery behavior of the community of cells between those endpoints following initial perturbations; ii) these assays do not reveal kinetics of such perturbations; iii) the monitored toxic endpoints may not include all mechanistic pathways leading to cellular toxicity; and iv) repeated ENMs dosimetry on cells is rarely done, missing a critical aspect of human exposure. Evidence is slowly accumulating that exposures to ENMs occur during different stages of production, handling, processing, and use of nanoproducts. Development of test systems capable of monitoring continuously and in real time communal behavior of cells over hrs to days using biologically-relevant global, label-free endpoints would alleviate several limitations of current cellular testing protocols.

Measurement in real-time of multiple physicochemical parameters implicated in ENMs toxicity is, at best, challenging, and often impractical. At present, the links between an ENM's physicochemical parameters and its toxicity are poorly understood and, therefore, it is not possible yet to predict ENM toxicity from the aggregate of these data. For these reasons, characterization of exposures to ENMs, especially as related to human health and the environment, and interpretation of their toxicological relevance, remains a major challenge. Expert panels have called for innovative approaches and technologies to tackle this complex problem [9,10]. Alternatively, biologically-relevant metrics, responsive to multiple key parameters involved in ENM toxicity, may provide an opportunity to screen for the inherent toxicity potential of ENMs and be adopted as novel ENM specific exposure metrics, providing a much needed link between common toxicological test systems (cells, animals) and human exposures. We believe that the living cell-based quartz crystal microbalance biosensor (QCMB) described below possesses unique technological features to fulfill the requirements described above and offers a great potential to serve the needs posed by nanotechnologies- the need for high rate toxicity screening and novel exposure dosimetry for human and environmental health studies.

### II. The Quartz Crystal Microbalance (QCM) as a live cell biosensor

For decades, piezoelectric quartz systems have been used in analytical chemistry because their oscillating frequency value, which can vary across the megahertz [(MHz), 10^6 ^cycles/sec] range, is sensitive and directly proportional to the crystal mass. Adding mass to the crystal lowers the oscillation frequency and decreasing mass raises it. This mass sensing ability has resulted in the creation of a nanogram mass sensing device known as the quartz crystal microbalance (QCM). Since the oscillating crystal detects any mass coupled to its surface, it has been widely used as a label-free signal transduction platform for a variety of chemical sensor and biosensor designs [11,12]. A number of more recent reviews of this platform have appeared in the application areas of drug discovery [13], clinical diagnostics [14], biochemical and cellular processes [15] and chemical biosensors [16-18]. More recently, cell based biosensors have been developed. In these devices, living cells are attached to the gold surface of the quartz crystal and serve as the sensing element, where cellular mass and viscoelastic properties affect the oscillation frequency of the crystal. Cells as the sensing element have the advantage that they possess a wide range of intelligent system properties resulting from the interplay of their integral membrane receptor-cytoskeletal-nuclear membrane systems to alter their mass distribution or viscoelastic properties due to external (i.e. cytokines, chemotactic agents, toxins, pathogens, chemical signals, pH or ionic alterations) or internal (i.e. DNA damage, mitochondria activity, cell polarity, new gene expression) signals. Thus, cells can detect a wide variety of changes in their environment, from individual molecules (traditional 'linear' biosensors) to more complex alterations (smart biosensors). These intelligent properties can be exploited when cells are used as biosensor detection elements.

In a series of studies developing cell based biosensors, we have demonstrated that cell attachment to an oscillating crystal could be studied to detect novel cell-cell cooperativity during the earliest stages establishing the cell based biosensor through cell coupling to the crystal surface [19-23]. Once formed, the cell based biosensor represented by a monolayer of cells attached to the crystal can detect cytokine initiated mitosis before any other conventional method [19]. Small molecules that affect specific aspects of the cells' cytoskeleton can be analyzed. Taxol and nocodazole, drugs that bind microtubules and hyperstabilize them vs. dissociate them, respectively, have opposite effects on the crystal oscillation frequency when using a cell based sensor [21-23]. We showed the nocodazole effect in the biosensor exhibited the log[effect] vs. dose response behavior of a typical drug with a midpoint of 0.9 μM. Thus, the biosensor was shown to be able to function as a drug discovery and characterization device. Advantages of live cell biosensors are that they can be used as continuous monitoring devices and can detect cumulative effects from low dose toxins. The other advantage of a biosensor technology in the evaluation of environmental exposures to ENMs is that it measures a direct effect of the nanomaterial on living cells and avoids the complications that ENMs may interfere with assays by disrupting or binding to assay reagents, may prevent or compromise the readout, or contaminant the equipment used for assays [5]. The output is gathered continuously and is automated so brief transient exposures are detected and the kinetics of cell stress and recovery can be measured. In this report, we describe experiments that demonstrate the ability of live cell based biosensors to detect and provide unique temporal response data following living cell exposures to single-walled carbon nanotubes (SWCNTs).

## Methods

### Cell lines and culturing conditions

The DH82 macrophage cell line was obtained from American Type Tissue Culture Collection (ATCC, Manassas, VA) and stock cultures were established and grown in media recommended by the manufacturers, at 37°C in a humidity controlled incubator with 5% CO_2_, unless otherwise noted. The media used was Eagle's Minimum Essential Medium (ATCC, Manassas, VA) supplemented with 15% heat-inactivated fetal bovine serum (FBS; Invitrogen, Carlsbad, CA), 200 mM L- Glutamine, 10 K IU Penicillin, 10 K mg/ml Streptomycin Sulfate (GPS Sigma, Saint Louis MOMO), and Amphotericin B (FGZ; Lonza, Basel, Switzerland). The macrophages were grown in 75 cm^2 ^rectangular canted neck cell culture flasks with vented caps (Corning Life Science, Lowell, MA), and re-fed every three days and passaged at least once a week in stock cultures.

### Phagocytic studies

Phagocytosis using living macrophages was analyzed using 3 μm diameter Zymosan A (S. cerevisiae) BioParticles^®^, Alexa Fluor^® ^488 conjugate (Invitrogen, Carlsbad, CA) or 0.8 μm polystyrene beads (Sigma, Saint Louis MO) vs. SWCNTs. Polystyrene microparticles are negatively charged stabilized colloidal particles. Source and characterization of SWCNTs is described below.

For phagocytic experiments, cells were trypsinized from stock flasks with 0.05% trypsin with EDTA (Invitrogen, Carlsbad, CA) and plated into either 8-well Lab-Tek™ II slide chambers (Nalge Nunc International, Rochester, NY), or into at least three QCM devices at equivalent cell densities (growth areas for Lab-Teks™ was 0.7 cm^2^/Well vs. growth area for QCM which was 0.4 cm^2^/Well. Cells were plated at 9.3 × 10^4 ^cells/LabTek chamber; and into each QCM as 5.0 × 10^4 ^cells/QCM device. Cells were then allowed to attach for 24 hours at 37°C in a humidity controlled incubator with 5% CO_2_.

After the 24 hr attachment period, duplicate Lab-Tek™ control wells or QCM wells were trypsinized to determine attachment frequency. Cells were counted in the overlaid media, a PBS wash and three consecutive trypsin cell removal steps. The standard of practice is that only when an 80% or greater attachment efficiency was obtained were QCM devices or Labteks used for experiments. The cell counts were also used to calculate a correct particle or bead concentration. For the Zymosan A experiments, a 1:100 cell to particles concentration was used. For example, approximately 7.44 × 10^6 ^beads (1:100) were added to 74,400 cells based on an 80% attachment efficiency from 9.3 × 10^4 ^plated cells in LABTEK experiments. For polystyrene bead experiments, a 1:100 cell to polystyrene beads concentration was used. These ratios were per manufacturer's recommendations and ideal for stimulating phagocytosis by macrophages and for photomicroscopy following cell ingestion. The remaining wells and QCM devices had media changed and the devices were then moved to room temperature and open ambient air to simulate more natural environmental monitoring conditions such as in a manufacturing or usage setting. For QCM experiments, after the attachment period and cell counting of a QCM device, the remaining QCMs moved to room temperature and open ambient air were connected to the automated computer data collection and crystal oscillator. Base line QCM traces were established for the next two hours and only if a stable frequency was observed, were QCM experiments continued. At least two QCM devices were run in parallel, a treatment QCM and at least one cell-containing QCM as an environmental baseline control response trace for the next 18 hrs. In some cases, additional QCMs with media were prepared in parallel without cells and were treated with either the highest dose of SWCNT alone, or with Zymosan A alone, or with polystyrene beads alone. These served as controls over the same time period as QCM cell experiments.

Cells in Lab-Tek™s were examined using phase microscopy and photographed at various times and fixed or stained as described below. At the end of all QCM experiments, cells numbers in the QCM were determined in the spent media, a PBS wash and in two to three consecutive trypsin cell removals using non-SWCNT treated QCM devices. This could only be done with non-SWCNT containing wells so as not to contaminate cell-counting equipment and other lab devices. These cell numbers were compared to the time zero cell numbers to detect loss of cells over the time course. This procedure and the gold QCM surface-cleaning procedure with successive PBS, water, ethanol and other washes were used to remove all residual extracellular matrix and cell debris, in order to regenerate the surface for the next experiment, and have been previously described by us [22,23].

### Fixation and staining of cells

For immunohistology, cells in Lab-Teks™ were first fixed by adding an equal volume of 4% paraformaldehyde (Electron Microscopy Sciences, Hatfield, PA) in PBS to the well containing media (in a 1:1 volumetric equivalent) for 10-15 min at room temperature. The cells were then fixed using 4% paraformaldehyde again, and then rinsed with PBS. The cells were then dual stained with a cytoplasmic stain, Lava Cell (Activemotif, Carlsbad, CA) and a DNA stain, 4',6-diamidino-2-phenylindole (DAPI). DAPI staining was performed by removing the PBS and replacing it with a 0.2 μg/ml solution of DAPI in methanol and incubating the cells at RT for 30 min. The cells were then rinsed with PBS, and the 24 μM Lava Cell in PBS was applied for 30 min RT. The cells were then rinsed with PBS; air dried, mounted with ProLong^® ^Gold (Invitrogen, Carlsbad, CA) and a cover slip applied. Images were obtained using fluorescence microscopy (Olympus, Center Valley, PA). Mitotic, apoptotic and interphase DNA morphology could be seen by DAPI as previously reported by us [24,25]. The DAPI images were taken using an excitation wavelength filter of 350 nm and the Lava Cell images were taken using an excitation wavelength filter of 534 nm. For the Zymosan A treated cells, the Zymosan A is viewed using an excitation wavelength filter of 480 nm.

### Photomicroscopy of SWCNTs inside live and fixed cells

For the SWCNT treated cells, a bright field image was taken. The image was then manipulated with Adobe Photoshop to render the SWCNT green. First the contrast & brightness was set for all images to 44, then images were inverted and the threshold adjusted to 128 to remove the artifact of cell membranes. Color was added by changing the hue and saturation. In the colorize mode, the hue was adjusted to 137, saturation to 77 and light to -50. To create a composite, the three images obtained (DAPI, Lava, SWCNT) were overlaid using DP manager version.3.1 software (Olympus, Center Valley, PA).

### SWCNTs

SWCNTs used in this study were purchased from Cheap Tubes Inc., (Brattleborough, VT, USA). These SWCNTs were synthesized via combustion chemical vapor deposition technique (CCVD) and were reported to have >90% wt purity. The preparation was reported to have >5%wt of co-produced multi-walled CNTs and >3%wt of amorphous carbon content as impurities.

### Physicochemical characterization of SWCNTs

**Figure 1:**
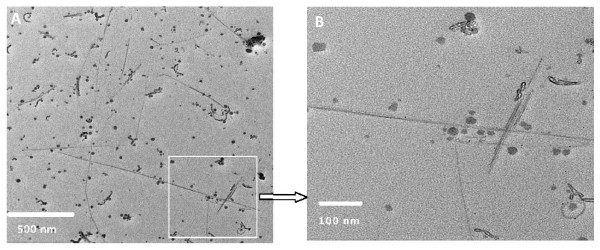
**Transmission electron micrograph (TEM) of the SWCNT dispersion in Triton-X 100 using probe sonication**. Dispersion efficiency was nearly identical in Eagle's medium, except that high salt and protein content made visualization of SWCNTs more challenging. The images in Triton-X are presented due to their visual clarity by TEM. Panel A-The dispersed CNTs appear as long straight needle-like nanofibers, varying in length from 0.5 to several microns and accompanied by spherical metal catalysts and organic impurities, better observed in the enlarged insert shown as panel B.

The physicochemical characterization of SWCNTs are summarized in Table 1. The properties of the SWCNTs include: specific surface area by BET (Brunauer, Emmett, and Teller N_2 _adsorption method); total and water soluble fractions of a panel of most important transition metals (i.e. Fe, Cr, Co, Ni, Mo, Mn,) determined using microwave-assisted acid digestion and ICP-MS based on the EPA 3051A method; organic carbon content was determined using a surrogate measure of organic material and the OC/EC ratio based on a modified NIOSH method 5040; surface charge in PBS saline; crystallinity by XRD, and morphology by TEM (Figure 1) described below. The polycyclic aromatic hydrocarbon (PAH) content of these SWCNTs was determined with the EPA Method 3546 (microwave extraction) and method 8276 (GC-MS). Additionally, we measured the biological oxidant damage potential and generation of reactive oxygen species (ROS) using the Ferric Reducing Ability of Serum (FRAS) [26] and using the modified DCFH assay [27].

**Table 1:** Physicochemical characterization of SWCNTs

SWCNT_L (Long Single-Wall CNTs)				
Primary particle size	Outer Diameter = 1-2 nm Length = 5-30 μm	Organic Carbon (OC, μg g^-1^)		19.4

Specific Surface Area (m^2^g^-1^)	510.50	Elemental Carbon (EC, μg g^-1^)		921.7

XRD spectra	Co [15-0806] present in the spectrum, consistent with the material description.	Organic Carbon/Total Carbon		0.021

**Dispersion efficiency in solution by DLS**		**Transition metals (ug/g, ppm)**		
		
			**Total**	**Water soluble**

Zeta Potential(mV)	-10.39	Cr	476	<0.1

Effective Particle Size (nm)	371.50	Co	1798	1.1
		
		Mo	1672	31

Electrophoretic Mobility (μm/s)/(V/cm)	-0.814	Mn	25.3	0.1
		
		Zn	4.1	1.3

Polydispersity Index	0.47	Ni	67.3	<0.1
		
		Fe	741	<0.6

The SWCNTs had high concentrations (μg/g) of Co (1798), Mo (1672), Fe (741) and Cr (476), and small amounts of Ni (67) and Mn [25]. The water soluble fractions of these metals were negligible (Table 1). The diffraction pattern (XRD) was consistent with its primary particle size, chemical composition, and purity levels from other analyses and Co was present in the XRD spectrum. The sample had 19.4 ppm (2.1%) of organic carbon. In addition, the SWCNTs used were found to have quantifiable levels of two PAHs (ppb): fluorene (95) and phenanthrene (189) with all other congeners being below the method limit of detection [28]. The SWCNTs were also found to have a significant oxidative damage potential in human blood serum-among the highest values tested in this assay for different classes of ENMs: 1,376 trolox equivalent units (TEUs, μmol L^-1^)/10 mg under the assay conditions, equivalent to 136.7 TEUs (μMol)/mg or 2.70 TEUs/m^2^. The SWCNTs were found to induce significant ROS generation in the DCFH assay as well, equal to 1812.8 (μM H_2_O_2 _eq./m^2^) at 0.3 mg/mL.

### Dispersion of SWCNTs

Dispersion of SWCNT solution for cellular testing was prepared in the actual culture medium to avoid possible re-agglomeration when stock dispersions of SWCNTs were spiked into the culture medium of a different composition. Dispersion of a stock solution of 1 mg/mL SWCNTs was based on a modification of published protocols (18-20), and involved using 1% BSA/Eagle's minimum essential medium and probe sonication for 10 min with 30 sec cycle at 30% amplitude (~200 W), while maintaining the scintillation vial in ice at all times. Dispersion efficiency was evaluated with Dynamic Light Scattering (DLS-Zetasizer, Malvern Instruments) and parameters of isolated SWCNTs were found to have a mean effective hydrodynamic diameter in solution of 371.5 nm (Table 1).

### SWCNT Transmission Electron Microscopic Characterization

The morphology of raw SWCNTs in the dispersions was evaluated using transmission electron microscopy on a Philips EM 400T for particle size and morphology [29]. Representative images in Triton-X 100 dispersion medium are shown (Figure 1). Dispersion efficiency of SWCNTs was nearly identical in both Triton-X and Eagles' media, a property confirmed with DLS measurements. However, the images of SWCNTs are much clearer in Triton-X due to lack of salts and proteins. The dispersed CNTs appear as long straight needle-like nanofibers varying in length between 0.5 to several microns (Figure 1). The insert image (Figure 1B) shows a magnified view of a single strand of SWCNT, surrounded by metal impurities. The length of CNTs is shorter than the vendor's specifications (5-30 μm) and our own measurements suggest fracturing of long SWCNT fibers during probe sonication.

### QCM methods and device

**Figure 2:**
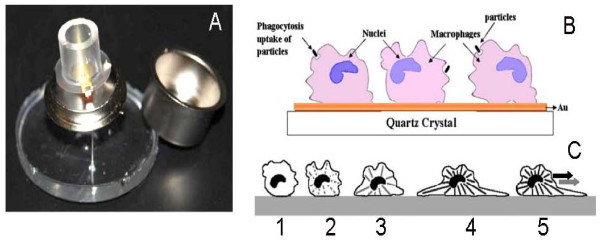
**The QCM device and schematics of the established and bioactive cell based biosensor**. We construct QCM devices using gold coated 10 MHz crystals and biocompatible materials for the growth of cells in a cylinder chamber filled with growth media, as described in the methods. The entire QCM device containing live cells can then be capped, as shown, for employment in non-laboratory settings and air samples perfused into the device in a controlled manner for periodic environmental testing (panel A). To create the living cell biosensor, macrophages are added to the growth media above the crystal and cells sediment down to and attach to the quartz crystal surface over a 24 hr period. When SWCNTs, polystyrene beads or Zymosan A are added to the media of stably attached cells within the QCM, the cells will phagocytose this material (panel B). These behaviors will be measured by changes in the crystal oscillation frequency. For example, in the act of attachment (1) and spreading (2-4), the cells will synthesize and polymerize cytoskeletal elements (panel C). Round cells will cause a maximal decrease in crystal frequency oscillation whereas with increased spreading and distribution of cellular mass, the crystal frequency will increase from steps 1 thru 4 and reach a level of homeostasis that is cell number dependent (panel C). Without perturbation, this frequency will remain stable. However, if macrophages are then stimulated to phagocytose, they will exhibit increased migration, involving re-rounding via de-polymerizing these cytoskeletal elements then re-polymerizing these monomers into highly aligned long elements to promote hyperextension of unilateral processes (step 5, panel C).

An AT cut quartz crystal of resonant frequency 9.97 MHz with gold electrodes (5 mm diameter) was used to create the base of a cylindrical well (Figure 2A). The well was created using a tube composed of Polydimethylsiloxane (Gelest Inc., Morrisville, PA) bonded to the surface of the crystal housing, forming a reservoir for the cells and media. PDMS, being a form of silicone rubber, was tested for cell compatibility and found to be non-toxic. The diameter of the cylinder exceeded that of the crystal itself to avoid loading the crystal directly under the electrodes and thus to have minimal impact on the resonant frequency. Prior to cell plating, the gold QCM surface was cleaned as described above before assembly in the well holder. The QCM device was placed within a large petri dish, filled with distilled water, and covered with a piece of PDMS, then placed inside a 37 degree temperature-regulated cell incubator for cell plating and attachment as described previously. After sterilization and washing with water and PBS, 200 μL of medium containing 50,000 DH82 cells was added to the well onto the QCM electrode and the entire holder was placed into the incubator with atmosphere controlled at 5% CO_2 _for 24 hr. Then the QCM was moved to ambient air and room temperature and the QCM electrode was plugged into a lever oscillator. At this point, the QCM device could be capped (Figure 2A) and perfused with air samples with a controlled periodicity.

A Tektronix TDS 2012B two channel digital storage oscilloscope (Tektronix) and lever oscillator (ICM, 35366-10), was used for the simultaneous computer-controlled measurement of the resonant frequency (f) as a function of time. For experimental treatments, the stable values at 2-3 hr were taken as baseline values before the addition of test materials. For direct additions of test samples, 50 μl of media is removed from the top of the QCM device and replaced with 50 μl of pre-warmed medium containing specific amounts of test materials or vehicle alone. Automatic monitoring of the f values at 1-min intervals was carried out using a PC and Labview software and the f values were continually automatically monitored. Frequency shift, Δf, plots were generated by subtracting measured f values from the baseline f before additions. Crystal oscillation is generated by a lever oscillator circuit (International Crystal manufacturing Co., Inc, Oklahoma City, OK) designed for driving a piezoelectric resonator under the heavily damped conditions associated with in-liquid operation. The frequency of the oscillation is measured via a fast Fourier transform and recorded once a minute. With a multi-channel oscilloscope, separate QCM's were able to be run in parallel allowing for control samples to be run under the exact same environmental conditions as treatment sample.

The frequency of crystal oscillation after cell addition is dependent on the macrophage attachment and degree of spreading on the surface of the crystal (Figure 2B and 2C). When first attaching as a round cell (panel C, step 1), the frequency will be maximally decreased. As cells spread, as is shown in steps 2-4, the cells using their cytoskeletal elements, will distribute their mass over a larger area, increase their viscoelasticity and the end result will be an increase in crystal oscillation to a homeostatic level relative to the cell attachment state. If stably attached macrophages are then stimulated to phagocytosis (panel B) they will exhibit increased migratory, cytokinetic and chemokinetic behavior as shown in step 5 (panel C). This can result in cyclic increases and decreases in crystal oscillation as cells alternatively round and hyperextend their cell body during directional mobility.

At the end of an experiment, the number of cells adhering to the QCM surface was determined by multiple trypsinizations and electronic cell counting. Trypsin treatment will remove cells and cellular protein from the crystal surface and the crystal oscillation will increase significant to pre-cell addition biosensor levels.

### LDH assays and standard curves with or without inclusion of SWCNTs

Lactate dehydrogenase enzyme activity in cell supernatants was measured with TOX-7 kit (Sigma Aldrich). Due to SWCNT binding to the enzyme [30,31], the calibration curve was performed with a pure LDH standard (Sigma Co., St. Louis) and in parallel using lysed cell solutions with different concentrations of SWCNT. Different numbers of cells (monosuspension) were plated in duplicate into Labteks for 24 hr at 37°C and 5%CO_2 _as described above. After DH82 were seeded in 8-well LabTeks for 24hrs, old media was replaced with fresh media with or without SWCNTs at final concentrations of 10, 30 or 100 μg/ml. Cells were then incubated at RT and ambient air for 24 hrs. The supernatants were collected into eppendorf tubes, and centrifuged at 250 g for 5 min at RT. These supernatants were then transferred to new tubes and centrifuged at 27,500 g for 60 min at 4°C to remove the SWCNTs. Supernatants were transferred to microfuge tubes for LDH assay. Lactate dehydrogenase assay mixture was freshly prepared for each experiment according to the manufacturers' instructions. Briefly, equal amounts of Lactate Dehydrogenase Assay Substrate, Assay Dye, and 1'x LDH Assay Cofactor were added together. 75 μl of the cell supernatant sample was diluted with 75 μl of PBS, so FBS became 7.5% in each tube and was added to 300 μL of the substrate/dye/cofactor mixture solution, light protected and incubated at room temperature for 30 minutes. 45 μl (1/10 volume) of 1 M HCl was then added to each tube to stop the reaction. Blank controls were prepared using a similar procedure as the above LDH assay except 1:1 diluted media was used instead of DH82 supernatant. Spectrometric absorbance was measured using a LAMBDA 35 UV/Vis Spectrophotometer (PerkinElmer, Inc.). Absorbance at a wavelength of 490 nm was subtracted by the absorbance readings at a wavelength of 690 nm. Each disposable cuvette was read in triplicate. The optical density values were then normalized to cell number using the calibration curves. Calibration curves with and without SWCNT effects were plotted by total lysis LDH reading versus total cell numbers derived from multiple trypsinizations and electronic cell counting in addition to the LDH pure standard curve.

The use of cell-derived LDH representing known numbers of macrophages allowed us to relate LDH units detected in the pure LDH assay to the actual number of lysed macrophages, as different cells types have different internal pools of LDH available. We also used the pure LDH standard in developing a calibration curve in the presence and absence of SWCNTs. By comparing cell-derived LDH to pure LDH with SWCNTs in the assay, we controlled for possible cell protein - SWCNT interactions that would alter the colorimetric readout using this assay.

## Results

### I. Non-toxic macrophage phagocytosis measured by QCM

**Figure 3:**
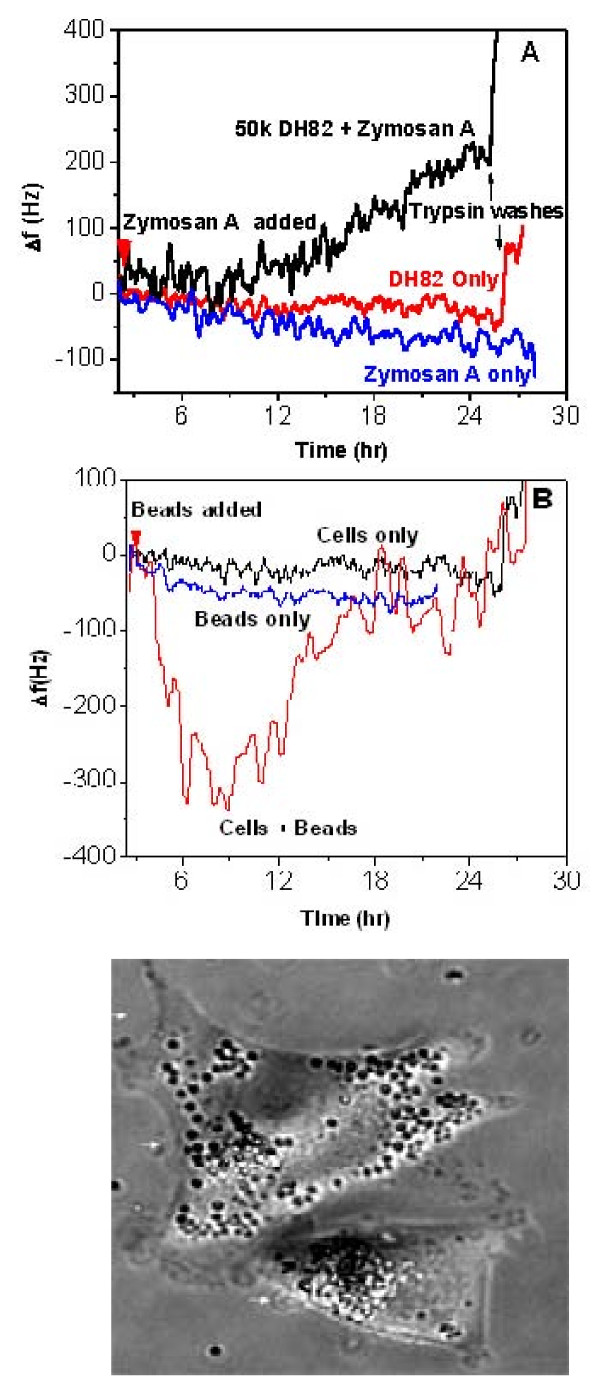
**Non-lethal macrophage phagocytosis measured by QCM**. Macrophage living cell QCM biosensors (50,000 cells/QCM) are shown operated at 10 MHz in parallel with control QCMs containing no cells (panels A and B). In experiments using degradable materials represented by Zymosan A (panel A), cells alone (red) and Zymosan A alone (blue trace) showed little change in frequency over 28 hrs, until trypsin cell removals (black arrows) at the termination of the experiment (panel A). Macrophages treated with Zymosan A (black trace) exhibited an increase in oscillation frequency that corresponds to increased migratory behavior by video-microscopy but frequency remains below 200 Hz indicating cells remain attached to the crystal, until trypsin cell removal (black arrow) and cell counting at the termination of the experiment (panel A). Cells are able to take up, degrade and regurgitate Zymosan A and it serves as a model system for macrophage neutralization of particulate material. As an example of macrophage phagocytosis of non-degradable materials, polystyrene beads were also used for testing (panels B and C). In the QCM, beads alone (blue trace) or cells alone (black trace) again led to little change in frequency over the 28 hr time course. In contrast, as living macrophages accumulated intact beads in their cytoplasm, as seen by phase microscopy (panel C), the frequency dropped over the first 12 hrs and then as cells regurgitated the beads, returned to baseline (panel B).

We first examined normal phagocytosis using Zymosan A (Figure 3A) or polystyrene beads (Figure 3B), classic methods for examining phagocytic processes, but never before studied using QCM.

For both types of experiments, the oscillation frequency was observed first for two hours under room temperature and ambient air conditions to determine its stability and was then normalized to a frequency shift value Δf of zero. This stable frequency represents macrophages that are attached but quiescent. At this point, one of the QCM devices was used for cell enumeration by counting cells in the media and trypsin washes of this QCM and viability determination was performed in the Cellometer (Nexcelom Bioscience, Lawrence, MA) using Trypan blue exclusion. At least an 80% plating efficiency was required and the cells needed to be 90% viable before commencing with additions in the QCM device. For studies with Zymosan A (Figure 3A), in some QCM devices only fresh media was then added as shown (red trace) and the frequency remained stable for the duration of the experiment, until trypsin cell removal was performed (small black arrow). In other QCM devices, not containing cells, Zymosan A was added (blue trace) at the same dose and time as that used for QCM devices containing cells (black trace at red arrowhead). Zymosan A alone (blue trace) showed a slight gradual decrease in frequency shift corresponding to crystals that had no cell layer on the crystal surface and a small increase in mass deposited on the surface of the crystal. However in contrast, QCM devices containing cells and receiving the same amount of Zymosan A exhibited a slight decrease and then an increase in frequency that never exceeded 200 Hz until trypsin cell removal (black trace, panel A black arrow). This change in frequency corresponds to the agitated migration of macrophages stimulated to collect and phagocytose Zymosan A, clearly contrasting with the frequency behavior of the unstimulated cells. This stimulated behavior requires the cells to spread, send out processes and attach leading edges of the cell body in a forward position while at the same time detaching the posterior side of the cell in order to exhibit directional mobility (Figure 2C). The cells do not detach or die in large numbers, as documented by the persistence of a slowly rising 200 Hz oscillation frequency, final cell numbers at the termination of the experiment, and the abrupt rise of the frequency exceeding 400 Hz when trypsin is finally applied (arrows) as cells detach from the surface. This behavior was confirmed in videomicroscopy experiments run in parallel.

**Figure 4:**
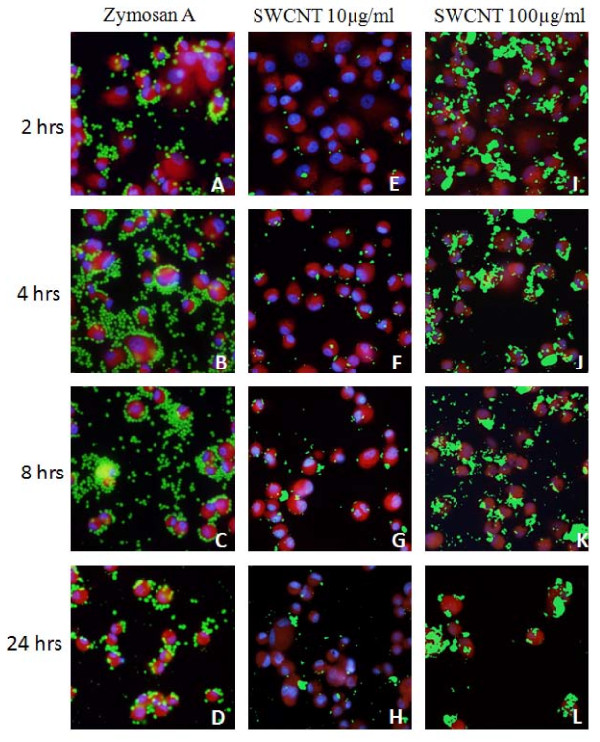
**Photomicroscopy of toxic and non-toxic macrophage phagocytosis**. As an example of phagocytosis and degradation by macrophages, Zymosan A was added to living cultures of macrophages and uptake, degradation and regurgitation by macrophage is shown at 2 (a), 4 (b), 8 (c) and 24 (d) hrs. DNA morphology of cells (blue) is normal in all these panels and reveals cells to be in either interphase or mitosis and cell numbers per field remains the same. At 24 hrs, degradation of particles of Zymosan A can be seen as regurgitated debris in the field. In contrast, when macrophages are treated with SWCNT (green; panels E-L) at low (10 μg/mL) and high (100 μg/mL) doses, the nanomaterial is seen to accumulate in the cytoplasm of the cells. At high doses of SWCNTs at 24 hrs (panel L), considerable loss of cell numbers from the surface is observed.

Macrophages gather and are able to degrade Zymosan A (Figure 4A-D) as described below. In contrast, macrophages can also be challenged to phagocytose non-degradable debris, simulated using polystyrene beads (Figure 3B). Again, after zeroing the baseline, cells alone (black trace) or beads alone (blue trace) over 18 hrs did not alter the oscillation frequency. However, when polystyrene beads were added to QCMs containing cells, these macrophages accumulated polystyrene beads within their cytoplasm (see Figure 3C) and could not degrade them. The oscillation frequency dropped 300 Hz by 12 hrs and then cells regurgitated the beads into the medium and recovered with the frequency shift returning to and exceeding baseline. These patterns of normal macrophage phagocytosis using degradable and non-degradable materials have been reported in the literature but have not previously been studied using QCM.

### II. Time course of phagocytosis of Zymosan A by macrophages

In Lab-Tek™ simulation experiments, macrophages were allowed to phagocytize Zymosan A at the same density as in the Figure 3 QCM experiments. At various times cells were fixed and stained (panels A-D). At 2 hrs (A), cells can be visualized using viable cell cytoplasm stains (red), DAPI to reveal A-T rich regions of DNA (blue), and Zymosan A (green) can be seen. By 4 (B) and 8 (C) hrs, cells have migrated to collect increasing amounts of discrete individual Zymosan A particles into their cytoplasm. Several mitotic cells are observed indicating that phagocytosis also stimulates cell division. By 24 hrs (D), the Zymosan A has been partially degraded by the cells and regurgitated, a part of the normal phagocytic process for biodegradable materials, and can be seen in the field as small fluorescent particulate material. DAPI staining in all panels of these figures indicate healthy normal interphase and mitotic cells (Figure 4) and confirms there is no change in vitality of the cells as a result of the phagocytic activity.

### III. Time course of phagocytosis of SWCNTs by macrophages

In parallel Lab-Tek™ simulation experiments (Figure 4), macrophages were treated with either low dose (10 μg/mL) SWCNTs (panels E-H) or high dose (100 μg/mL) SWCNT (panels I-L). Macrophages could be seen with concentrated ingested SWCNT (green). The cell density at 24 hrs (panel H) was slightly decreased to that at 2 hrs with low dose SWCNTs (panel E vs panel H). In contrast, after addition of 100 μg/ml SWCNTs, cells were observed to have ingested SWCNT material (green) at 2, 4, and 8 hrs but by 24 and 48 hrs there was significant loss of cells (red) from the culture dishes (L). By microscopy, zymosan particles are discrete and rapidly degraded by the cells over 24 hrs. In contrast, SWCNTs initially are observed as smaller aggregates on the surface and inside macrophages and cannot be degraded or released from the cell, eventually accumulating and causing death (panel L).

### IV. Macrophage response to various doses of SWCNTs as measured by QCM

**Figure 5:**
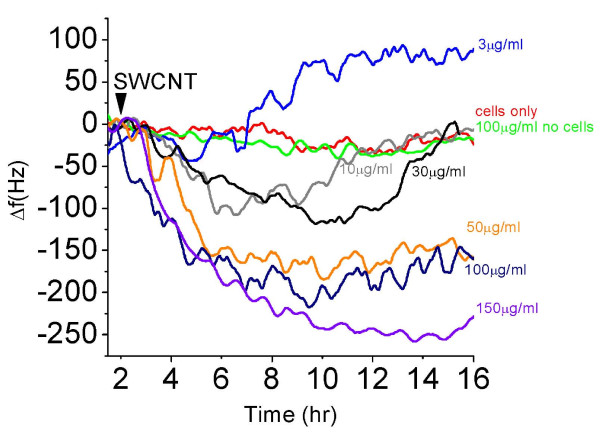
**QCM monitoring of macrophage responses to increasing doses of SWCNT**. Time and dose-dependent responses of living macrophage based QCM biosensors to varying doses of SWCNTs is shown. In separate experiments, the different doses of SWCNTs were added to the cells after a 24 attachment to the crystal surface. In each case the frequency shift, Δf, was determined from the recorded data and is plotted. This is the frequency difference between that value at the time of addition (arrowhead) and all subsequent times in the QCM response. Macrophage responses to 3 (blue), 10 (grey), 30 (black), 50 (yellow), 100 (navy) and 150 (violet) μg/ml SWCNT exposures are shown. Two control experiments are shown as well: QCM devices with macrophages and vehicle alone (1% BSA) (red); and the QCM, with no cells, responding to the addition of 100 μg/ml (green). In all cases, the frequency response traces represent the average of at least triplicate experiments.

Next, we carried out extensive macrophage biosensor experiments over a range of doses from 3-150 μg/ml SWCNTs in the QCM (Figure 5). Cells alone (red) or SWCNTs alone without cells at 100 μg/ml (green) caused no change in oscillation frequency. Low dose, 3 μg/ml SWCNT treatments of macrophages in the QCM, caused a slight decrease of oscillation frequency over the first five hours post-addition and then exhibited a steady increase of oscillation frequency until reaching 100 Hz (teal) in a pattern that resembled that of non-toxic phagocytosis seen with Zymosan A in Figure 3A. Ten (grey) and 30 μg/ml (black) SWCNT treatments lead to a significant additional decrease in oscillation frequency that was prolonged beyond the first five hours and we believe represents a form of cyto-stress. The QCM frequency then returned to a value characteristic of a non-lethal phagocytic response. In simulations, cells appeared to enlarge and exhibit numerous processes during this phase of cytostress, but did recover. Higher doses of SWCNT, 50 μg/ml (light green), 100 μg/ml (navy) and 150 μg/ml (violet), lead to a significant and prolonged decrease in crystal oscillation frequency that never recovered by 16 hrs.

### V. Signature QCM patterns of macrophage responses to various doses of SWCNTs and demonstration of phagocytosis and apoptosis in these cells

**Figure 6:**
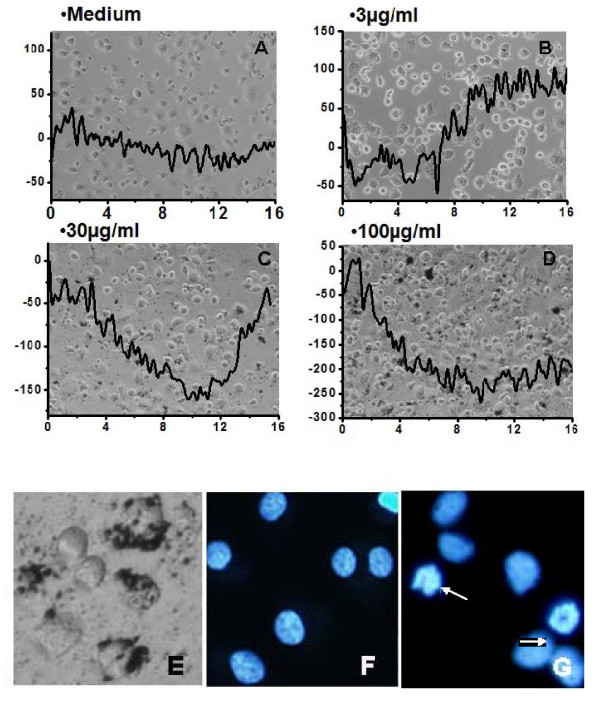
**Signature patterns of macrophage QCM responses to SWCNT exposures**. Time dependent QCM measured responses of live macrophages to vehicle alone (1% BSA)(A), low dose (B), cytostress-doses (C) and high (D) doses of SWCNTs. By 6-16 hrs, a signature pattern of four types of outcomes can be recognized that predicts observations in 24 hr and 48 hr simulations: no response, a normal phagocytic response, a cytostress response with cell recovery and a response followed by a toxic outcome, respectively. In panel E, washed live macrophages are shown 48 hrs after addition and uptake of SWCNTs (100 μg/ml). At 24 and 48 hrs, DNA staining confirms evidence of apoptosis in these treated cells (G) compared to untreated cells (F).

Four signature patterns of SWCNT treatment in the QCM emerge from these studies (Figure 6A-D). No treatment (A) has a stable frequency over 16 hrs; non-toxic treatment exhibits a small decreased frequency over the first 6 hrs and then a recovery higher than the original baseline indicative of agitated macrophages(B). Higher doses of SWCNT, exhibit a prolonged decrease of frequency and a slower recovery to baseline (C). And high dose treatments exhibit a decrease of frequency that never recovers(D). In Labtek simulations, accumulation of SWCNTs can be observed in the living cells using phase microscopy at 48 hrs (Figure 6E), similar to the non-degradable polystyrene bead accumulation see in Figure 3C. Lastly, condensed nuclei and two apoptotic bodies are seen at the high doses of SWCNTs by fluorescence microscopy at 24 and 48 hrs indicative of cell death by apoptosis (Figure 6G), compared to untreated cell nuclei (Figure 6F) as has been reported in the literature [32].

**Figure 7:**
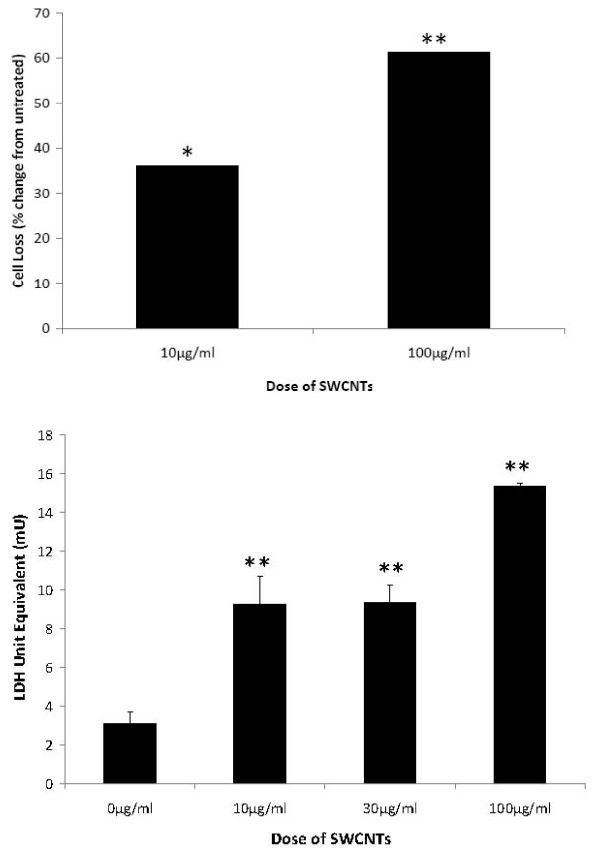
**Cytotoxicity of SWCNTs as a function of dose**. At 24 hrs, the number of cells remaining attached in five fields of each treatment group in duplicate was determined and statistically analyzed. A significant reduction in viable cells expressed as the percent cell loss (panel A) was seen as a function of SWCNT treatment in a dose response fashion. In separate experiments, lactate dehydrogenase (LDH) was measured in the growth media from living DH82 cell cultures in the presence or absence of SWCNTs over a 24 hr period using a quantitative assay. DH82 cells (81,600) cultured alone released LDH over a 24 hr period equivalent to the death of 7,000 cells (~9%), estimated from the standard curve performed in parallel without SWCNTs. With treatments of 10 or 30 mg/ml SWCNTs added to the cultures, statistically significant increased amounts of LDH were detected in the supernatant indicating 17-18,000 cells had died, or 22% of the cells. This was statistically increased above baseline. After treatment with 100 μg/ml SWCNTs, 28,000 cells died or 34%. These results were compared to a pure LDH standard performed using the same assay in the presence and absence of SWCNTs. Values are mean ± SD with three doses of SWCNT. Statistically significant differences from control are indicated with **; p < 0.001.

In simulations, we counted the number of cells that remained viable at 24 hrs after vehicle or SWCNT treatments using 10 or 100 μg/ml doses. In response to 100 μg/ml treatments we detected statistically significant loss of cells from the Lab-Tek™ surfaces, indicative that a cytotoxic effect was seen (Figure 7A). We also used a modified LDH quantitative assay to evaluate cytotoxicity of cells due to SWCNT treatments. First, we had to standardize the assay as nanomaterial can interfere with colorimetric assays. We took known numbers of DH82 cells (5K, 10K,15K, 20K, 25K, and 30K). We subjected this precise number of cells to total lysis. Then we divided these samples into 4 separate sets. Buffer alone was added to set 1,10 μg/ml SWCNTs to set 2, 30 μg/ml SWCNTs to set 3, and 100 μg/ml to set 4. These sets were then each read at 490 nm and 690 nm. The values were then calculated by subtracting the 690 nm from the 490 nm as recommended by the manufacturer and used to generate a standard curve of LDH readings for 5-30 K DH82 cells for each dose of SWCNT. In addition we performed an LDH standard curve using pure LDH in the presence and absence of each dose of SWCNTs. Using these standard curves, the data was highly reproducible and we evaluated loss of cells as measured by LDH release 24 hours after control or SWCNT treatment (Figure 7B). We found over 24 hrs a low level basal loss of cells, 9.8% of the total culture. In contrast treatment with either 10 or 30 μg/ml SWCNT resulted in a statistically significant loss of cells above baseline, equivalent to 22.3 and 22.4% of the population. Lastly, treatment of DH82 cells with 100 μg/ml killed ~ 35% of the culture as measured by 24 hrs (Figure 7B). These data are consistent with measured loss of enumerated cells from the growth surface shown in Figure 7A and predicted by the QCM profiles at 16 hrs (Figure 5). The role of transition metals or other components of SWCTs in the cytotoxicity is not yet known [33].

### VI. SWCNT treatment at non-lethal doses vary in their cell recovery times

**Figure 8:**
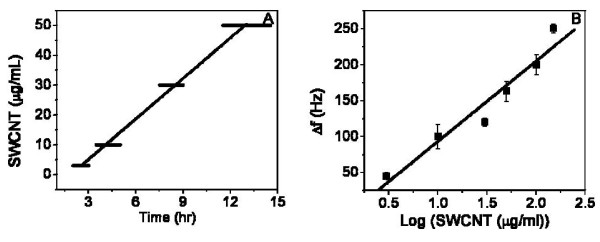
**SWCNT dose dependent frequency plots**. Panel A presents the SWCNT concentration vs. the time range required for Δf reversal from decreasing to increasing Δf. Only the lowest SWCNT concentrations: 3, 10, 30 and 50 μg/ml exhibited this reversal. Since the reversal was a shallow trough, a range is indicated for each SWCNT concentration on the x-axis. Nonetheless, a linear fit well described this dependence, as is shown. In panel B, the total maximum Δf decrease observed at each SWCNT concentration is plotted vs the log (SWCNT). Again, a linear fit well describes this dependence, as is shown and the SD are indicated.

We noted two additional properties of these dose dependent QCM Δf data that needed further examination (Figure 8). The first was a property of the initially decreasing Δ f trend reversing at various times observed for the four lowest low-lethal doses of SWCNTs. We took the time point interval of the broad maximum Δ f decrease (the length of time to the reversal following SWCNT addition) and plotted it vs. SWCNT concentration, as shown in Figure 8. Since the time of Δ f maximum decrease occurred over a broad range for these shallow response curves, we made the width of the reversal time points at each dose reflect the entire reversal time range in the plot (Figure 8A). While the points are broad, the behavior is clear; a linear trend was fit well to the data. Other curve fittings produced poor results. Therefore, we believe that this linear response between SWCNT dose and the interval before reversal occurs is revealing something fundamental about the cellular response to the SWCNT toxicity. A putative mechanism to explain this cellular behavior could be as follows. The mass of SWCNTs taken up into the cells via phagocytosis is linearly proportional to the dose of SWCNTs in the media. The linearly increasing SWCNT mass taken up requires a linearly increasing length of time to carry out detoxification of the SWCNTs up to ~50 μg/ml. When this process is complete and if the cells are not overcome by cytoxic effects, the Δ f reversal can occur. Above 50 μg/ml, SWCNT, the cells could not overcome these cytotoxic effects.

The second property we examined was the maximum Δf decrease produced by the different doses. We present these data in Figure 8B as a Δf vs. log[SWCNT] dose plot. The linear fit to these data are quite good. The functional dependent of these dose response data resemble the small molecule drug response we observed for different cell types in previous published QCM biosensor work for nocodazole, a microtubule dissociating drug [21,22].

In conclusion, these studies indicate that a real-time live-cell biosensor is able to provide unique information about the SWCNT exposures and reversibility of its toxic effects. The effect of repeat dosing at low SWCNT concentrations can now be studied to determine a cumulative effect. With a successful air sampling collection interface, the QCM biosensor may also offer unique opportunities for real-time monitoring of exposures to SWCNTs, and for utilizing its outputs (e.g maximum or mean Δf, lag time, etc.) as alternative metrics for studies of human health effects.

## Discussion

The biosensor consists of macrophages attached to the surface of a QCM and the health of the cells is reflected in the QCM crystal oscillation frequency which responds to the degree of cell coupling to the crystal surface, level of cell spreading and the cell's viscoelastic properties. Macrophages were used as the living cell sensing element, mimicking the cell in the lung involved in both clearance and retention of inhaled nanoparticles [34-36]. Two forms of normal phagocytosis were tested in parallel to represent a non-toxic baseline. QCM traces and simulation experiments run in parallel were performed using vital dyes to compare cell responses to Zymosan A, and polystyrene beads (both benign) vs. SWCNT (cytotoxic, from 3- 150 μg/ml). We found that low SWCNT concentration phagocytosis, 3 μg/ml, was indistinguishable from normal phagocytic QCM traces and the cells remained viable in parallel simulations. The 30-50 μg/ml SWCNTs experiments lead to a delayed or prolonged decrease of oscillation frequency before the QCM trace returned to a non-lethal phagocytic pattern. In simulations, low level cytotoxicity was detected 24 and 48 hrs later but 78% of cells recovered. High SWCNT treatment doses, 100-150 μg/ml, lead to a significant decrease in crystal oscillation frequency that never recovered at 16 hrs and in simulations, toxicity in the form of apoptosis and cell loss, occurred and could be measured at 24 and 48 hrs. Therefore, the QCM traces contained complex behavioral information of the community of cells, reflective of their activity and continued well-being.

These studies indicate that a real-time live-cell biosensor provides unique detection information of peak and toxic SWCNT exposures, and cellular response kinetics, not readily obtainable with standard assays. We believe these features of the QCM are applicable to the broad family of ENMs. The new biosensor has the potential to be a powerful tool in the field because it is relatively inexpensive, highly portable (the size of a credit card), can be operated in series to test multiple materials simultaneously, and can send real-time data remotely. Without continuous monitoring of a living cell response to sublethal exposures we would not have detected different indications of cytostress. Do exposed cells that recover have a memory of ECM exposure and are there long term effects of this exposure? Only in such a system can we challenge these cells again, vary the dose and the periodicity of exposure to address these types of questions and successfully assess cell vitality and even transformation potential. Repeated exposures are the norm in the real world, and repeated chronic low dose effects are relevant toxicologically but such measurements are nonexistent for ENMs. The QCM is superior to existing cellular testing platforms for being able to conduct such experiments in the laboratory.

Engineered nanomaterial (ENM) use has increased as different applications have been developed. However, during ENMs manufacturing or use they are often aerosolized; yet in many cases their safety when inhaled is unknown [37]. The use of macrophages in our cell based biosensor allows us to simulate the cell implicated in the lung retention and clearance following inhalation of ENMs. Our results are consistent with the report of Jia et al (38) using single-walled and multi-wall carbon nanotubes (CN) and guinea pig alveolar macrophages. These investigators found the uptake and toxicity of single or multiwalled CNs was much higher that that caused by fullerenes (C_60_). The single-walled CNs impaired phagocytosis and damaged subcellular structures of the macrophage at doses as low as 0.38 μg/cm^2 ^[38]. Evaluation of potential toxicity of ENMs continues to present a serious challenge, caused in part by the overwhelming variety of different types of ENMs as well as variations within the same ENM class. The complex interplay of different physicochemical properties of nanoparticles presents unique challenges for the common cellular test systems, through adsorption of dyes on nanoparticle surfaces, complexation of metals with dyes and fluorescence quenching, as well as light scattering effects. The SWCNT material used in our study has a purity of ~ 90% wt and several transition metals (Table 1). In its most widely used form, carbon nanotubes produced as powders for electronics, composite materials and optics usually do contain transition metals that act as catalysts during synthesis. In our future studies we are comparing the response of macrophages to carbon nanotubes with different geometrics and chemistries. In the current work and that of other investigators, the assessment of carbon nanotubes with transition metals is a realistic scenario for human exposures (37,38]. A label-free living cell quartz crystal microbalance (QCM) biosensor offers unique opportunities to provide continuous monitoring of cellular responses without these common drawbacks of cellular testing.

Validation of the biosensor against animal inhalation experiments is planned for the near future. The biosensor described has the potential to function as a personal dosimeter for human exposure assessment. The biosensor can provide an integrated biologically relevant response to the complex interplay of an ENMs exposure profile, its intrinsic toxic potential, and the ENMs' aerosol reactivity at the point of generation. For example, loss of toxicity due to aging of reactive aerosols cannot be captured in current post-sampling characterization. Additionally, the biosensor can respond to ENM mixture aerosols in the workplace and environment and can 'sense' their potential interactive/synergistic effects, and integrate these effects over broad particle size distributions relevant to human inhalation exposures. Similar to the use of biomarkers as surrogates for internal tissue dose in human epidemiological studies and toxicity evaluations, the biosensor response can be seen as a surrogate biomarker of ENM exposures. The reported study can contribute to a broad-based, high rate toxicity screening approach for ENMs; provide mechanistic information about ENM effects on living cells by providing dynamic data on the health and behavioral status of the community of cells; and be a unique tool in exploring dosimetry issues in real-time.

## Abbreviations

ENM: Engineered nanomaterial; QCMB: quartz crystal microbalance biosensor; SWCNTs: single-walled carbon nanotubes; QCM:quartz crystal microbalance; DAPI: 6-diamidino-2-phenylindole; CCVD: combustion chemical vapor deposition technique; PAH: polycyclic aromatic hydrocarbon; ROS: reactive oxygen species; FRAS: Ferric Reducing Ability of Serum; TEUs: trolox equivalent units

## Competing interests

The authors declare that they have no competing interests.

## Authors' contributions

GW built all the QCM devices used and carried out the QCM studies; AD carried out the phagocytosis simulations using polystyrene beads and zymosan and performed immunocytochemical staining of cells using fluorescent microscopy studies after SWCNT addition to macrophage cultures; JZ carried out the LDH and apoptosis assays and phase microscopy studies after SWCNT addition to macrophage cultures. AP and DB prepared all SWCNT samples, performed all characterization studies and TEM of SWCNTs. MV designed and built the electronics for the QCM devices. SJB drafted the manuscript, and participated in the conception and design of all the studies and supervised all simulation experiments. KM helped to design the QCM experiments, interpret the data and performed statistical analysis on the data. JT supervised all the QCM experiments, designed the QCM device and the electronics associated with the QCM. All authors read and approved the final manuscript.

## Authors' information

JT, KM and SJB are principal investigators of the work. JT is a electrical engineer with expertise in nanoscale sensors, nanoelectromechanical devices, chemical/biological sensors, optoelectronics, and standards of practice in nanomanufacturing. KM is a chemist with expertise in data mining of large biological, biomedical and molecular datasets and in characterization of polymer based bio materials and drug effects using biosensors. SJB is a cell biologist with expertise in radiation biology, regenerative medicine, nanotherapeutics and biosensors. DB is a chemist with expertise in nanomaterials, human health and safety, environmental monitoring and dosimetry.
